# Effect of Grazing-Mediated Dimethyl Sulfide (DMS) Production on the Swimming Behavior of the Copepod *Calanus helgolandicus*

**DOI:** 10.3390/md11072486

**Published:** 2013-07-15

**Authors:** Mark N. Breckels, Nikolai W. F. Bode, Edward A. Codling, Michael Steinke

**Affiliations:** 1 School of Biological Sciences, University of Essex, Colchester CO4 3SQ, UK; E-Mails: ecodling@essex.ac.uk (E.A.C.); msteinke@essex.ac.uk (M.S.); 2 Plymouth Marine Laboratory, Prospect Place, The Hoe, Plymouth PL1 3DH, UK; 3 Department of Mathematical Sciences, University of Essex, Colchester CO4 3SQ, UK; E-Mail: nbode@essex.ac.uk

**Keywords:** chemical ecology, chemical interactions, marine plankton, dimethyl sulfide (DMS), *Calanus helgolandicus*, copepod behavior

## Abstract

Chemical interactions play a fundamental role in the ecology of marine foodwebs. Dimethyl sulfide (DMS) is a ubiquitous marine trace gas that acts as a bioactive compound by eliciting foraging behavior in a range of marine taxa including the copepod *Temora longicornis*. Production of DMS can rapidly increase following microzooplankton grazing on phytoplankton. Here, we investigated whether grazing-induced DMS elicits an increase in foraging behavior in the copepod *Calanus helgolandicus*. We developed a semi-automated method to quantify the effect of grazing-mediated DMS on the proportion of the time budget tethered females allocate towards slow swimming, typically associated with feeding. The pooled data showed no differences in the proportion of the 25 min time budget allocated towards slow swimming between high (23.6 ± 9.74%) and low (29.1 ± 18.33%) DMS treatments. However, there was a high degree of variability between behavioral responses of individual copepods. We discuss the need for more detailed species-specific studies of individual level responses of copepods to chemical signals at different spatial scales to improve our understanding of chemical interactions between copepods and their prey.

## 1. Introduction

Interactions mediated by bioactive infochemicals operate across a range of temporal and spatial scales in marine environments, influencing population dynamics, community structure and ecosystem functioning [1]. These bioactive compounds affect fundamental and diverse processes including food selectivity, prey encounter and capture, mating interactions, chemical defense, behavior and population synchronization [2]. The climate-relevant trace gas dimethyl sulfide (DMS) is formed following the cleavage of the temporally abundant algal secondary metabolite dimethylsulfoniopropionate (DMSP). Several studies have indicated that airborne DMS from marine plankton is a bioactive compound that acts as a directional foraging cue to a range of vertebrate predators enabling them to locate high productivity prey patches [3,4,5,6]. The process of DMS-cleavage is accelerated following viral lysis, natural senescence and grazing. Steinke *et al.* [7] showed that the copepod *Temora longicornis* can detect gradients of DMS and responds with a change in its swimming behavior. Since copepods can effectively regulate phytoplankton and microzooplankton populations (e.g., [8,9]) their ability to react to grazing-induced DMS could be an important process in structuring pelagic ecosystems. Ubiquitous in the marine environment, volatile DMS provides an ideal model for investigating chemically-mediated trophic interactions in plankton communities [10]. Recently, Ferrer and Zimmer [11] suggested that DMSP and DMS are “molecules of keystone significance”, which, analogous to the keystone species concept, have the potential to initiate major trophic cascades and structure marine communities. This is supported by mathematical simulations that suggest that DMS and other infochemicals are potentially important in structuring planktonic multitrophic interactions [10]. Despite the potential ecological significance of bioactive chemical interactions structuring planktonic communities, the behavioral responses of planktonic predators to grazing-induced volatiles remains poorly resolved [2,12].

Phytoplankton succession leads to small DMS-producing species dominating phytoplankton assemblages during the summer [13]. Copepods, such as *Calanus helgolandicus*, feed inefficiently on small phytoplankton [14] and preferentially prey on microzooplankton, which form a crucial trophic bridge between primary producers and higher trophic levels [15]. Grazing by microzooplankton on DMSP-containing phytoplankton has been shown to result in rapid production of DMS both in laboratory and field experiments [16,17]. Under such conditions, DMS has the potential to mediate trophic interactions across planktonic trophic levels. Copepods display considerable behavioral plasticity in response to chemical and hydromechanical stimulation, and these behaviors may be modified or habituate with time after initial exposure [18,19]. The ability for copepods to detect and modify their behavior in response to grazing-induced DMS production may enhance their foraging success.

The copepod *Calanus helgolandicus* employs a “hop and sink” swimming strategy involving a period of swimming followed by a period of inactivity when the copepod sinks. These predominant behaviors are interspersed with rapid grooming events and escape reactions [20]. During slow swimming *C. helgolandicus* generate a large laminar feeding current drawing prey towards the mouth appendages. Maintenance of the copepod feeding currents is not considered to be energetically expensive [21,22], but in the absence of prey stimuli, breaking will allow negatively buoyant copepods to sink slowly through the water column until further detection of prey. As generation of the feeding current causes hydrodynamic disturbances to a volume of water up to 180 times the volume of the copepod [23,24], breaking in the absence of prey cues allows copepods to remain less conspicuous to potential predators. As such, copepod behaviour is a trade-off between optimising encounters with prey whilst reducing energetic costs and susceptibility to predation [25,26]. Such behavioral trade-offs are likely linked to perceived prey density and the perceived threat of predation [27].

Detection of copepod responses to prey stimuli has previously been measured in two ways, including through changes in the frequency of appendage beating or the proportion of the time budget allocated towards slow swimming. Gill and Harris [28] observed significant differences in the beat frequency of the first maxilla in *C. helogolandicus* in response to dinoflagellate and diatom diets in comparison to seawater controls, but the observed differences were typically small. Conversely, large differences in the proportion of the time budget spent slow swimming were observed between some dinoflagellate diets (*Prorocentrum micans* 11.5% and *Scrippsiella trochoidea* 28.4%) and the diatom treatment (*Thalassiosira weissflogii* 38.9%). However, variation in the proportion of the time budget spent slow swimming was high [28]. Here, we used a semi-automated technique to test the hypothesis that DMS generated following microzooplankton grazing increases the amount of time copepods spend slow swimming. We quantified the behavior of tethered *C. helgolandicus* feeding on the heterotrophic dinoflagellate *Oxyrrhis marina* that was pre-conditioned with either a high or low DMS-producing strain of the phytoplankton *Emiliania huxleyi*.

## 2. Experimental Section

### 2.1. Cultures

Two non-calcifying strains of the coccolithophore *Emiliania huxleyi*, a high DMS-producing strain (CCMP 373) and a low DMS-producing strain (CCMP370) were purchased from the National Centre for Marine Algae and Microbiota (NCMA) and maintained in batch culture in ESAW medium [29] at 15 °C and a light intensity of 100 μmol m^−2^ s^−1^ during a 12 h light: 12 h dark cycle. The heterotrophic dinoflagellate *Oxyrrhis marina* (CCAP 1133/5) was cultured in filtered (0.2 μm poresize) and autoclaved seawater (FASW), and maintained on a diet of non-DMS producing *Dunaliella tertiolecta* (grown in ESAW as above). Following grazing by *O. marina*, CCMP 373 produces 15-fold higher DMS concentrations than CCMP 370 [16]. Prior to experiments, *O. marina* was placed in the dark for 24 h to eliminate prey. Copepods (*Calanus helgolandicus*) were collected weekly by trawling with a coarse mesh cod-end at the Western Channel Observatory sampling site L4 off Plymouth, U.K. (50°15′ N, 04°13′ W). Copepods were transported to the laboratory in insulated cool boxes before adult females (average prosome length = 2.21 ± 0.119 mm) were microscopically identified and transferred into 20 L buckets containing FASW in a dark walk-in room at 15 °C. They were maintained on a diet of *Rhodomonas salina* and *O. marina* at approximately 200 μg C L^−1^.

### 2.2. Tethering

Adult females were tethered to a hair 24 h prior to experiments. Hairs were treated with acetone and washed with Milli-Q water to remove natural odors. The hair was glued to the end of a glass Pasteur pipette which had been heat molded into a position allowing copepods to be oriented in a natural swimming position. During tethering, copepods were placed ventral side down in a concaved microscope slide with the feeding appendages and antennae immersed in water. The dorsal prosome was gently dabbed dry with microscope cleaning tissue and the hair glued to its base using instant adhesive (3M Scotch-Weld CA4OH). Tethered copepods were transferred into filtered seawater and fed *O. marina*. Tethered individuals typically survived for several weeks and copepods used in experiments were maintained for a minimum of 24 h after the experiment to ensure survival before freezing at −80 °C for storage and body measurements.

### 2.3. Filming Set-Up

Filming was carried out in a dark controlled-temperature room at 15 °C. Copepods were attached to a micromanipulator and placed in 1 L polycarbonate bottles filled to the neck with FASW. A CCTV camera (Watec, WAT-902H) with a 60 mm macro lens (Nikkor) was connected to a computer to record copepod behavior. Illumination was provided using collimated light from an infrared light-emitting diode (peak wavelength 940 nm).

### 2.4. Treatments

Polycarbonate bottles were inoculated with either *E. huxleyi* CCMP370 (low DMS) or CCMP373 (high DMS) to achieve a density of 6000 cells mL^−1^. *Oxyrrhis marina* was added to achieve a final density of 350 cells mL^−1^. Bottles were placed on a plankton wheel at 1 rpm and incubated for 5 h to allow grazing interactions to occur and the build-up of DMS. A total of 16 copepods were exposed to each treatment for 45 min using the following treatments in sequence: FASW, initial treatment diet, FASW, alternate treatment diet. The order of treatment (low DMS and high DMS diet) was randomized to prevent potential bias. The experimental design was based around a repeated measures approach allowing comparisons of individual copepods behavior between high and low DMS treatments.

### 2.5. DMS Quantification

DMS measurements were taken from 5 mL samples, gravity filtered through a 25 mm GF/F filter into a narrow glass vial minimizing diffusive losses. Depending on the expected DMS concentration in the sample, 1 to 4 mL was immediately transferred from the bottom of the vial to a purge-and-trap system for cryogenic enrichment of DMS before quantification using gas chromatography (Varian 3800) with a pulsed flame photometric detector (PFPD) [30]. Each experimental replicate was sampled in analytical duplicate.

### 2.6. Behavioral Analysis

**Figure 1:**
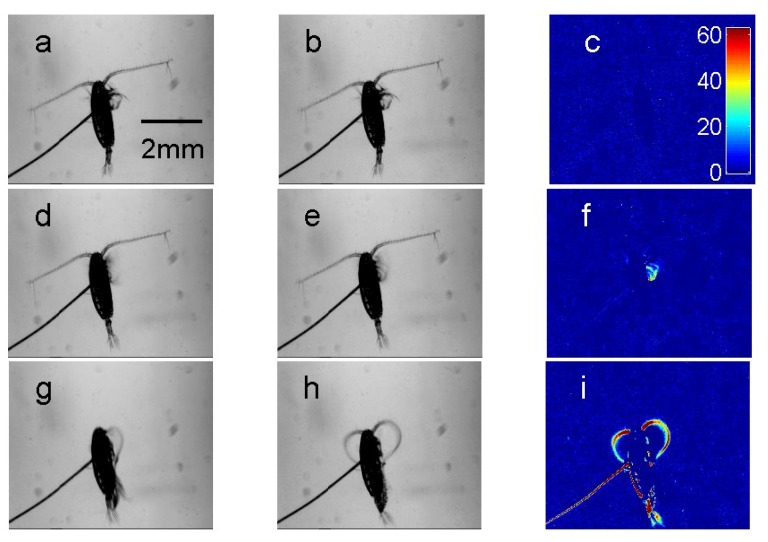
Consecutive frames from video recordings of behavioral states of a tethered copepod with associated color-coded maps for changes in pixel intensity. Image **a** and **b** show two consecutive frames during copepod breaking resulting in minimal pixel change **c**. Images **d** and **e** show two consecutive images recorded during slow swimming. The pixel color intensity around the swimming appendages can be observed in image **f**. Images **g** and **h** show two consecutive images during grooming, this behavior resulted in large changes in pixel intensity. The color scale is identical across **c**, **f** and **i**.

**Figure 2:**
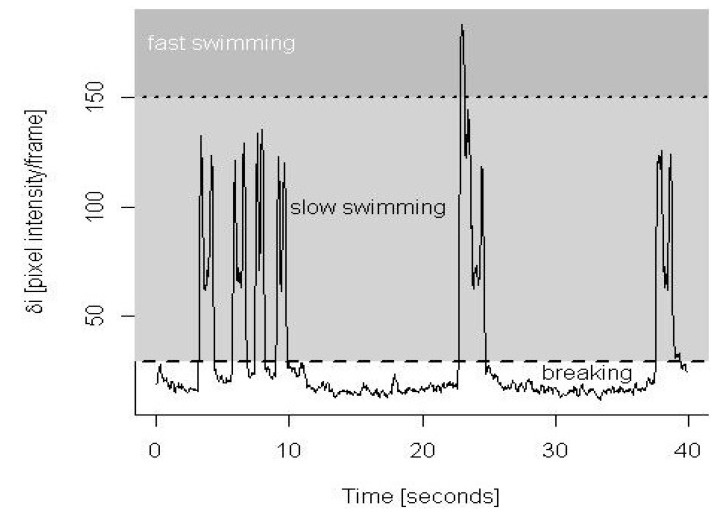
Semi-automated method for classifying copepod behaviors. From video recordings of tethered copepods we extracted maximal changes in pixel intensity (δ_i_) over time. For each individual copepod, threshold values were assigned to identify periods of breaking (no shading), slow swimming (light grey shading) and fast swimming/grooming events (dark grey shading). The spikes in δ_i_ at the start and end of slow swimming bouts were due to the movement of the tether (a flexible hair) at the onset and end of copepod movement. In between these spikes, δ_i_ took lower values resulting from the copepod’s moving appendages (see Figure 1f). A video showing the slow swimming bouts and grooming behavior associated with this 40 s sequence is available as supplementary material online.

A semi-automated technique was developed to assign copepod behavior into three states: breaking, slow swimming or fast swimming (including escape reactions and grooming see Figure 1 and supplementary material). A custom MATLAB code subtracted the pixel intensities of two consecutive grey-scale video frames and determined the maximum change in pixel intensity, δ_i_, across the matrix obtained from this subtraction. Since copepods were tethered, no movement (breaking) led to small changes in pixel intensity, continuous rhythmic feeding appendage movements (slow swimming) led to intermediate changes in pixel intensity and rapid movements of the body (fast swimming) led to large values in δ_i_. Subsequent analysis of δ_i_ time series was conducted in the R programming language [31]. To avoid erroneous classifications due to short-term fluctuations in δ_i_, we smoothed these time series by averaging δ_i_ over a five-frame window. By visual comparison of the δ_i_ time series to observed behavior in our video recordings, we determined two thresholds for each film: one to distinguish breaking from swimming and another to distinguish slow from fast swimming (Figure 2). Using these thresholds, we then converted the δ_i_ time series into time series of behavioral states adopted by the copepods. To ensure the robustness of this approach, we visually compared the automated classification for each film to the behavioral states observed in our video recordings at three time points over 1000 frames at the beginning, middle and towards the end of the video sequences. In order to gain an estimation of error, the automated method was compared to a previous data set that had been analyzed manually frame-by-frame (see results). Of the total 16 copepods filmed, the data from four copepods were discarded due to difficulties in assigning behavioral threshold values in at least one of the treatment videos. The behavioral state of 12 copepods was extracted for a period of 25 min from the beginning of recordings. Analysis over this period of time (37,500 frames) allows observation of behaviors at the onset of each treatment and is sufficiently long enough to allow copepods to habituate to the treatment conditions [19]. Due to the time taken to position the copepod and focus the camera system between replicates, analysis commenced approximately 1 min after initial transfer. Behavioral states were recorded at a temporal resolution of 25 frames s^−1^.

### 2.7. Statistical Analyses

Percentage data on the copepod behavioral time budgets was arcsine transformed and tested for normality. *t-*tests were initially used to determine if there were any differences in the average duration of time spent slow swimming in each treatment depending on the order they were received. The effect of DMS treatment and time was tested by analyzing each minute of behavioral data for the 12 copepods over the 25 min time interval of the experiment using 2-way repeated measures analysis of variance in SPSS Statistics 18 setting the threshold for α at 0.05. Differences in the proportion of the time budget allocated towards slow swimming between the two treatments were also tested at the individual level. The difference between the means of paired observations for slow swimming percentage over the 25 min time interval were compared by testing confidence intervals with matched pairs analysis for small sample sizes [32].

## 3. Results and Discussion

### 3.1. Validation of Behavioral Analysis

A semi-automated approach was developed to test the hypothesis that grazing-mediated DMS production would stimulate an increase in the proportion of time *Calanus helgolandicus* spent feeding. We used three videos from a previous study [33] to verify the semi-automated analysis of copepod swimming behavior. Each video was 15 min in length and each of the total 67,500 frames had been manually scored for the different behavior types. The semi-automated approach consistently overestimated the proportion of the time budget allocated to slow swimming by an average of 3.7 ± 0.48% of the total time. This error may be due to the necessary smoothing of data and was considered acceptable given the significant decrease in time required for analysis. Since improvements of our optical set-up have been made since the previous study, improved image resolution will have resulted in an estimated error of <3.7% in our analysis of slow swimming behavior. The method allowed efficient analysis of tethered copepod behavior at a high temporal resolution for extended video sequences compared to laborious manual analysis by eye. A disadvantage of the method was that all “fast swimming” behaviors were pooled, as escape reactions and grooming events could not be differentiated in our analysis. However, these behaviors typically accounted for less than 1% of the behavioral time budget of all copepods in our experiments.

### 3.2. DMS Quantification

Grazing by *Oxyrrhis marina* on the low DMS-producing strain of *Emiliania huxleyi* (CCMP370)resulted in concentrations of 1.8 ± 0.67 nM DMS in the low DMS treatments after 5 h incubation. Grazing by *O. marina* on the high DMS-producing strain of *E. huxleyi* (CCMP373) in the high DMS treatment resulted in significantly higher DMS production with final concentrations of 13.1 ± 4.30 nM (*t* = 13.46 d.f. 21, *p* < 0.001). Filtered seawater used in the experiments had a natural DMS concentration of 1.2 nM (±0.75).

### 3.3. Pooled Copepod Behavior

**Figure 3:**
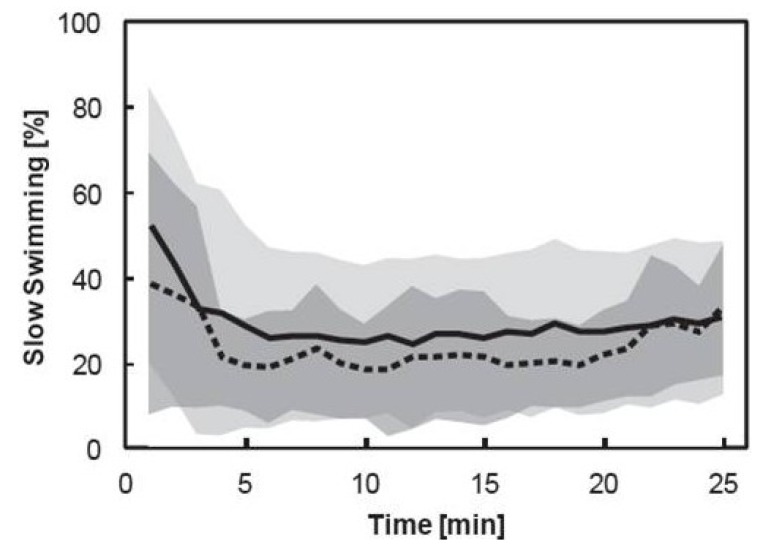
The proportion of the pooled time budget copepods allocated towards slow swimming in high (dashed line) and low (solid line) dimethyl sulfide (DMS) treatments. Values represent the mean of 12 copepods each minute for 25 min of analysis. Standard deviation of the mean is shown as the dark grey shaded area for the high DMS treatment and light grey shaded area for the low DMS treatment.

Slow swimming and breaking behaviors accounted for the majority of the copepod behavioral repertoire. Fast swimming behaviors, including escape reactions and grooming events, made up the remaining time budget and accounted for less than 1% of the 25 min time budget across all copepods and treatments. Treatment order did not influence the average time spent slow swimming during the 25 min period of analysis. Copepods spent the same proportion of the total time budget slow swimming irrespective of the order they received high (*t*_1,10_ = 0.44, *p* = 0.667) or low DMS treatments (*t*_1,10_ = 0.10, *p* = 0.924). There were no significant differences in the average proportion of the 25 min time budget the 12 copepods allocated towards slow swimming in the high (23.6 ± 9.74%) and low (29.1 ± 18.33%) DMS treatments (Figure 3, Table 1). The behavior of the copepods changed significantly with time throughout the duration of the 25-min experiments in both treatments (Table 1), with pooled data showing that copepods spent more time slow swimming at the onset of each treatment (Figure 3). Copepods have previously been shown to increase swimming and escape reactions immediately in response to treatment perturbations but such behaviors can be reduced through treatment habituation during the 25 min treatment and filming [18,19]. In our experiments there was no significant interaction effect between DMS treatment and time (Table 1).

**Table 1:** Results of a repeated measures ANOVA test showing the effect of high and low DMS treatments, and time on the proportion of the time budget copepods spent slow swimming. The behavior of 12 copepods was analyzed for 25 min, with each minute being tested in the model.

	**d.f.**	**MS**	**F**	***p***
DMS	1	0.727	2.11	0.177
Error (DMS)	10	0.354		
Time	24	0.112	3.88	<0.001
Error (Time)	240	0.029		
DMS × Time	24	0.015	1.45	0.087
Error (DMS × Time)	240	0.100		

### 3.4. Individual Copepod Behavior

**Figure 4:**
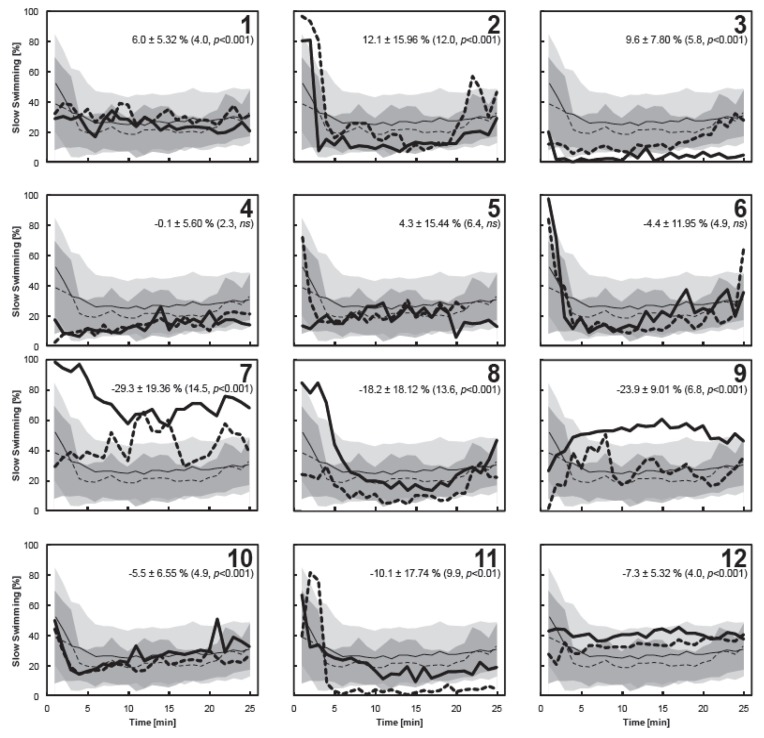
The proportion of the time budget 12 individual copepods allocated towards slow swimming in high (thick dashed line) and low (thick solid line) DMS treatments. Each individual is displayed against the pooled data for the high and low DMS treatments with standard deviations shown as the shaded area as described in Figure 3. Values associated with each panel show the mean difference (±S.D.) between the paired treatments calculated from matched pairs analysis with the test statistic (*t*α_/2_) and the significance level (*p*) given in brackets; a positive value indicates copepods spent more time slow swimming in high DMS treatments (statistically significant in Copepods 1–3). Where the value is negative, copepods spent significantly more time slow swimming in Low DMS treatments (Copepods 7–12). Data for the last 4 min of the video sequence is missing for Copepod 5 in the high DMS treatment due to movement of the copepod out of the plane of camera focus.

Individual copepods varied in their behavioral responses at the onset of each treatment (Figure 4). Matched pairs analysis showed that three of the twelve individuals (copepods 1–3) spent significantly more time slow swimming in high DMS treatments, whereas six copepods (7–12) spent significantly more time slow swimming in low DMS treatments (Figure 4). Differences in the proportion of the time budget dedicated to slow swimming between the mean of matched pairs analysis from the high and low DMS treatments was checked against the differences in measured DMS concentration between the paired treatments but no trend was observed (data not shown). Whilst there were no clear trends in the proportion of time spent slow swimming by *C. helgolandicus* in response to DMS, a large degree of variability in the time budget allocated towards slow swimming was observed between copepods at the individual level irrespective of DMS concentration (Figure 4). For example, Copepod 7 spent a large proportion of the total time budget slow swimming in the low DMS treatments (72.5%) whilst Copepod 3 spent just 3.9% of the time budget slow swimming in the same treatment. Whilst it is important to consider that the results from these experiments are drawn from a sample size of 12 copepods and are subject to experimental noise, they do raise questions regarding the variability of behavior. An interesting avenue for future studies is to determine the potential existence of individual “personalities”, as described for invertebrate behaviors [34] and recently revealed in decapod crustaceans [35]. Such differences in individual behavior within a population may diminish the effects of changes to the environment as not all individuals react in the same fashion [36]. Future studies should consider repeated exposure of the same individual to a particular experimental treatment to provide more detailed insight into the degree of behavioral variation in ecologically important copepods.

Pelagic copepods are taxonomically diverse and display various swimming and feeding strategies. A range of environmental cues transcending various scales are utilized to maximize copepod foraging success [37]. The relative importance of bioactive compounds relative to other cues in determining copepod foraging success is likely to be constrained by species-specific feeding ecology. From the perspective of a foraging copepod, bioactive infochemicals such as DMS, potentially operate across three broadly defined scales: (1) the micro or cellular scale where cellular exudates may provide copepods with an advanced warning of an approaching prey item; (2) the meso or patch scale where background chemical cues could elicit feeding associated behavior modifications; and (3) the macro or water column scale where chemical gradients may provide directional cues to locate high productivity ephemeral prey patches. Our experiments exposed *C. helgolandicus* to a productive prey patch with either a high or low DMS signal. In such a scenario at least two of the above mechanisms could operate to provide copepods with chemical foraging cues. Below we discuss our results in light of previous findings on the response of copepods to chemical cues and in relation to species-specific feeding ecology and ecologically-relevant scales.

The ability of copepods to utilize chemical cues at the microscale is highly constrained by swimming strategy. For example, ambush predators such as *Oithona similis* do not utilize prey exudates in the boundary layer, and instead rely on hydromechanical signals to detect prey [38]. Cruise-swimming and current-generating copepods, such as *C. helgolandicus*, have been suggested to use remote chemical detection from the deformation of the boundary layer surrounding cells entrained in their feeding currents [39,40,41,42]. However, recent studies have questioned the theory of remote prey detection suggesting that prey exudates are only effective at much shorter distance [43,44]. Tiselius *et al.* [44] filmed encounters of two copepods (*Pseudocalanus* sp. and *Paracalanus parvus*) with various prey items, and determined that chemically-mediated remote sensing does not occur. In addition, by modeling the boundary layer chemical concentration, Tiselius *et al.* [44] conclude that remote sensing could only occur for “very large or unusually leaky cells”. It remains to be established if rapid volatile production, such as the liberation of DMS following microzooplankton grazing, constitutes sufficient chemical exudation allowing remote chemical detection by copepods.

At the meso- or patch scale, elevated background concentrations of infochemicals may stimulate an increase in foraging-related behaviors. This has been observed in copepods in response to amino acid additions (e.g., [45]) and cell-free prey filtrates [28]. In contrast to the findings from earlier experiments using DMS microinjections into the flow-field of tethered *T. longicornis* [7], our experiments with *C. helgolandicus* suggested that grazing-mediated DMS has no clearly defined and consistent effect on swimming behavior when considering data pooled across all 12 individuals. *C. helgolandicus* spent approximately a quarter of the time budget dedicated to slow swimming in the low and high DMS treatments. This is consistent with the proportion of the time budget spent slow swimming by *C. helgolandicus* feeding on a range of dinoflagellate diets and in filtered seawater, whilst diatom prey resulted in an increase in swimming [28]. The presence of *Oxyrrhis marina* in our experiments may provide a sufficient hydromechanical stimulus indicating the presence of a prey patch, potentially overriding the additional DMS cue in the high DMS treatment. Other studies have found copepod behavior to be closely coupled to prey density, with copepods in filtered seawater spending very little time slow swimming [18]. Van Duren and Videler [25] demonstrated that the copepod *Temora longicornis*, a typical “cruise-swimmer” (in contrast to the “current-generating” behavioral strategy displayed by *C. helgolandicus*), modifies its swimming speed depending on prey density in accordance with predictions of optimal foraging theory. Woodson *et al.* [37] showed that copepods narrow their search area through a series of cue hierarchies. It is likely that the physical sensing of prey from hydromechanical cues or direct encounters provides copepods with cues for area-restricted search or feeding behaviors, whilst infochemicals provide a more general cue for copepods to display extended search behaviors [37]. In a series of experiments designed to uncouple the relative importance of hydromechanical and chemical cues in copepod foraging behavior Buskey *et al.* [46] showed that the behavior of *Pseudocalanus minutus* differed depending on the cue received. In the presence of prey *P. minutus* decreased swimming speeds and increased turning rates, whereas cell-free prey exudates elicit an increase in swimming speed combined with a decrease in turning rate [46]. Increasing the relative tortuosity of swimming trajectories within a prey patch would increase residency time allowing copepods to exploit the area of high productivity. Whilst increases in swimming speed and reductions in turning rate in response to prey cues, resulting in more ballistic swimming trajectories, would increase the rate of encountering a prey patch [47]. It is possible that chemical cues such as DMS operate at larger scales than tested in our multitrophic interaction experiments, allowing *C. helgolandicus* to exploit directional cues to locate high productivity prey patches. For example, swarming behaviour in response to specific dissolved amino acids has been observed in laboratory experiments with the copepods *A. hudsonica* and *Eurytemora herdmani* [48]. However, once a copepod encounters a high productivity prey patch hydromechanical cues appear to become the overriding foraging cue. Further experiments are required to determine the response of copepods to DMS and other bioactive compounds in heterogeneous chemical environments and to elucidate the interplay between prey density and infochemicals on feeding behavior.

## 4. Conclusions

We tested the hypothesis that grazing-induced DMS production following microzooplankton herbivory on phytoplankton would result in increased feeding behavior, measured in terms of time budget allocation towards slow swimming, in the copepod *Calanus heloglandicus*. Pooled data from 12 individual copepods over a 25-min period in treatments with either high or low grazing-induced DMS showed no difference in the total amount of the time budget allocated towards slow swimming.

Individual level responses showed a large degree of variation in between-individual behavior, both in terms of the total amount of time allocated towards slow swimming between copepods and in terms of the response of individual copepods to DMS treatments. Six of the twelve copepods showed a significant increase in slow swimming in low DMS treatments whereas only three copepods increased the proportion of slow swimming in high DMS treatments. This result highlights a cautionary note for assuming “average” copepod behavior and adds to the growing argument of the existence of individual behavioral “personalities” in invertebrates.

Our experiments replicate the scenario of a copepod encountering a high productivity prey patch with the addition of either high or low grazing-mediated DMS cues. In light of previous work and our results, we suggest that copepod behavioral responses to potential grazing-induced infochemicals, such as DMS, may act over larger spatio-temporal scales. Further work to determine the scale-dependent role of DMS as a chemical cue for copepods is required.

## Supplementary Files

Supplementary File 1 Supplementary File (WMV, 9995 KB)
